# Trends in Clinical Features of Primary Hyperparathyroidism During 2013–2024

**DOI:** 10.1155/ije/9936658

**Published:** 2026-01-07

**Authors:** Yuansi Chen, Junhe Wang, Shuo Li, Qinying Zhao, Kunling Wang, Ming Liu

**Affiliations:** ^1^ Department of Endocrinology and Metabolism, Tianjin Medical University General Hospital, Tianjin, China, tjmugh.com.cn; ^2^ Tianjin Institute of Endocrinology, Tianjin, China

**Keywords:** asymptomatic PHPT, clinical features, PHPT, primary hyperparathyroidism

## Abstract

**Objective:**

Clinical features of primary hyperparathyroidism (PHPT) differ between developed and developing countries. In past decades, patterns of PHPT have changed while there are currently few data on large cohorts in Chinese. Therefore, this study aims to describe changes in clinical features of Chinese patients with PHPT.

**Materials and Methods:**

685 patients with PHPT were collected and divided into several subgroups by time periods, symptoms, and pathological types. Clinical characteristics were compared among subgroups.

**Results:**

Patients were divided into 177 cases (25.8%) in group A (2013–2018) and 508 cases (74.2%) in group B (2019–2024). Compared with group A, parathyroid hormone (PTH) was significantly lower and clinical manifestations were milder, and the percentage of asymptomatic patients was higher in group B. Bone pain (46.8%), nephrolithiasis (37.3%), and fatigue (36.2%) were the most common symptoms in symptomatic patients. Serum PTH, calcium, osteocalcin, and urine pH levels in symptomatic patients were higher than those in asymptomatic patients, while serum phosphate and bone density levels were lower. In addition, among 417 surgical patients, benign parathyroid tumor was in 373 cases (89.4%), atypical parathyroid tumor was in 34 cases (8.2%), and parathyroid carcinoma was in 10 cases (2.4%). Compared with benign PHPT, serum PTH, calcium, alkaline phosphatase, and bone turnover marker (BTM) levels were significantly increased, and serum phosphate level was decreased in malignant PHPT.

**Conclusion:**

Clinical features of PHPT changed during 2013–2024 with remarkably increasing asymptomatic PHPT patients, whose clinical manifestations were milder, and complications were less.

## 1. Introduction

Primary hyperparathyroidism (PHPT) is a prevalent endocrine disorder characterized by excessive secretion of parathyroid hormone (PTH), leading to significant complications. With the development of serum calcium examination from 1970s, the incidence of PHPT in the United States and Europe has been on the rise, with estimating that it affects approximately 2 in 1000 individuals [1, 2], particularly among middle‐aged and elderly women. At the same time, an asymptomatic profile has been appeared via such method mentioned above. Majority of patients in western countries demonstrate no symptoms with a mild elevation in serum PTH and calcium [3]. Though the prevalence of asymptomatic PHPT increases these years, PHPT in China still exhibits a symptomatic pattern [4, 5].

The complications associated with PHPT include osteoporosis, fractures, nephrolithiasis, and cardiovascular diseases, all of which can severely impact patients’ quality of life [6]. Current clinical management of PHPT primarily involves parathyroidectomy (PTX), which is effective in alleviating symptoms and preventing complications. However, many patients present with complications at the time of diagnosis, and a notable proportion of individuals remain asymptomatic, leading to underdiagnosis and delayed intervention, ultimately resulting in adverse outcomes. Therefore, understanding clinical characteristics and trends of PHPT is crucial for improving diagnostic and therapeutic strategies in clinical practice.

This study aims to analyze clinical and biochemical features of patients with PHPT and to explore the trends over different time periods. Additionally, it seeks to compare the differences between asymptomatic and symptomatic PHPT patients and also the differences between benign and malignant PHPT.

## 2. Materials and Methods

### 2.1. Patients

Our study collected people with PHPT who were admitted to our institution from January 2013 to December 2024. The diagnostic criteria refer to the guidelines for diagnosis and treatment of PHPT [7]: (1) inappropriately evaluated or uninhibited PTH level with the presence of hypercalcemia (both defined by the mean of two measurements); (2) exceptions for secondary hyperparathyroidism and familial hypocalciuric hypercalcemia. The diagnosis of asymptomatic PHPT was based on lack of classical symptoms or signs which were traditionally associated with hypercalcemia or PTH excess [6]; diagnosis was made occasionally by serum calcium examination or neck ultrasonography. The study followed the Helsinki Declaration and was approved by the ethics committee of our institution (IRB2024‐YX‐310‐01). No informed consent was required for this study.

### 2.2. Materials

Hospitalization information of people with PHPT was collected from the electronic medical record system. All patients were referred to hospital according to symptoms or abnormal examinations, and fasting blood and morning urine were retained. Disease duration was defined as the time from the first detection of symptoms or abnormal examinations to looking for medical treatments. Standard laboratory methods for measurements of plasma and urine calcium, creatinine, and phosphate were used. Serum calcium = (40 − plasma albumin level (g/L)) ∗ 0.02 + measured serum calcium levels (mmol/L) [7]. Plasma intact PTH levels were measured using a chemiluminescent method. 25‐OHD levels were analyzed by chemiluminescent particle immunoassay. Osteocalcin (OC), type I collagen carboxy‐terminal peptide (CTX) and type I procollagen amino‐terminal prepeptide (PINP) were measured by electrochemical luminescence method. Urine pH levels were measured by urine analysis strip (dry chemical method), which type was MEDITAPE UC‐11A. eGFR was calculated according to the MDRD correction simplified formula = 175 ∗ ((Scr (μmol/L)/88.4)^−1.234^) ∗ (age (years)^−0.179^) ∗ gender index (male = 1, female = 0.79) [8]. Body mass index (BMI) = weight in kg/height in m squared (kg/m^2^).

All patients underwent ultrasonography of neck and urinary system. Bone density examination: The bone density of lumbar L1–L4, femoral neck, and whole hip was determined by dual‐energy X‐ray absorptiometry (DXA) method. Low bone state was diagnosed in women before menopause and men less than 50 years old with *Z* value ≤ −2.0. Osteoporosis was diagnosed in postmenopausal women and in men ≥ 50 years of age with *T* value ≤ −2.5, and osteopenia was diagnosed with −2.5 < *T* value < −1.0 [9].

Surgical indications [6] were as following (including those who are asymptomatic): (1) serum calcium > 0.25 mmol/L above the upper limit of normal or (2) bone mineral density (BMD) by *T* value ≤ −2.5 or (3) eGFR < 60 (mL/min ∗ 1.73 m^2^) or (4) nephrocalcinosis or nephrolithiasis by imaging modality or (5) age < 50 years.

The histological diagnosis of parathyroid carcinoma is restricted to a parathyroid neoplasm that shows one of the following: full‐thickness capsular invasion, vascular invasion, perineural invasion, or invasion into adjacent structures. Atypical parathyroid tumor is defined that demonstrates atypical cytological and architectural features but lacks unequivocal capsular, vascular, or perineural invasion or invasion into adjacent structures or metastases reflecting a parathyroid neoplasm of uncertain malignant potential [10].

### 2.3. Statistical Analysis

The Statistical Product and Service Solutions 22.0 (SPSS 22.0) was used to process the data. Non‐normal distribution continuous data were represented by median and upper and lower quartiles [*M* (Q25, Q75)]. Nonparametric tests were employed for group comparisons. Categorical data were expressed as composition ratio *n* (%), and Chi‐square test was used for intergroup comparison. *α* = 0.05 was used as the statistical significance threshold.

The specific strategies for nonparametric tests and correction for multiple comparisons were as follows: Mann–Whitney *U* method and Bonferroni correction were used for comparison between two groups. Kruskal–Wallis *H* method was used for comparison among multiple groups and Holm‐Bonferroni correction was used for all pairwise as post hoc multiple comparisons.

Multivariate logistic regression analyses were used to explore the relationship between various indicators and the presence of symptoms and the pathological type, respectively. The results were shown as odds ratios (ORs) and 95% confidence intervals (CIs). The analysis of each independent variable adjusted age, gender, and BMI.

## 3. Results

### 3.1. Demographics and General Features of Patients With PHPT

Our study included 685 patients: 166 (24.2%) were males, 519 (75.8%) were females (male‐to‐female ratio of 1 : 3.1), 464 (67.7%) were symptomatic, and 221 (32.3%) were asymptomatic.

In recent years, the number of PHPT patients in our hospital had gradually increased (Figure 1(a)). In particular, the number of those with asymptomatic PHPT has developed greatly, from 4 in 2013 to 44 in 2024. The population of total PHPT markedly increased by 5.7 times from 2013 to 2024. PHPT was most commonly observed in patients aged 50–69 years old (Figure 1(b)).

Figure 1(a) Number of patients with primary hyperparathyroidism (PHPT), asymptomatic PHPT, and symptomatic PHPT from 2013 to 2024. (b) Age distribution of patients with PHPT.

**Figure:**
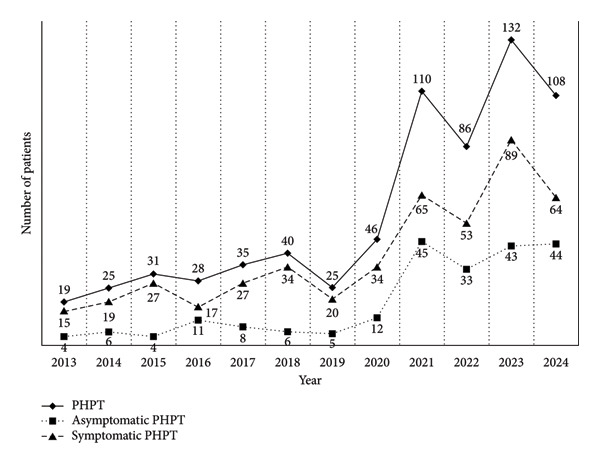
(a)

**Figure:**
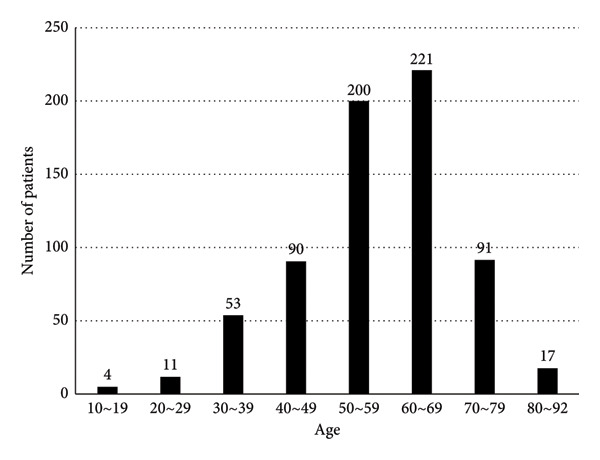
(b)

### 3.2. Clinical and Biochemical Features of PHPT

The most frequent manifestations of symptomatic patients were bone (49.8%), including bone pain (46.8%) and fracture (9.3%); urinary system (44.0%), including nephrolithiasis (37.3%), polydipsia and polyuria (7.5%); and muscle (39.2%), including fatigue (36.2%) and muscle pain (4.7%) (Table 1).

**Table 1:** Clinical manifestations of the symptomatic PHPT patients (*n* = 464).

Manifestation	Number (%)
Bone	231 (49.8)
Bone pain	217 (46.8)
Fracture	43 (9.3)
Urinary system	204 (44.0)
Nephrolithiasis	173 (37.3)
Polydipsia and polyuria	35 (7.5)
Muscle	182 (39.2)
Fatigue	168 (36.2)
Muscle pain	22 (4.7)
Digestive tract	173 (37.3)
Nausea and vomiting	163 (35.1)
Constipation	21 (4.5)
Gastrointestinal ulcer	5 (1.1)
Neuropsychiatric system	65 (14.0)

Abbreviation: PHPT = primary hyperparathyroidism.

In asymptomatic PHPT patients, 54.3% were identified through laboratory testing of serum calcium during routinely health checkup or examination for other diseases, while an additional 22.6% were detected via conventional neck ultrasound examination and the rest of 23.1% were identified as PHPT by both examinations above.

In total, 417 patients with PHPT met the criteria for surgery and underwent PTX and 80.3% (335/417) had parathyroid adenoma and, in five patients, the parathyroid glands were ectopic: three were in the anterior mediastinum, one was in the left tracheoesophageal groove, and one was beside the thymus gland. A total of 9.1% (38/417) of the patients had multiglandular parathyroid diseases, 8.2% (34/417) had atypical parathyroid tumor, and 2.4% (10/417) had parathyroid carcinoma (Table 2).

**Table 2:** Pathology of PHPT patients between 2013 and 2024 (*n* = 417).

Pathology	Number (%)
Multiglandular parathyroid diseases	38 (9.1)
Parathyroid adenoma (including ectopia)	335 (80.3)
Atypical parathyroid tumor	34 (8.2)
Parathyroid carcinoma	10 (2.4)

Abbreviation: PHPT = primary hyperparathyroidism.

We divided these 12 years into two periods, group A (2013–2018) and group B (2019–2024), to investigate the patterns of change in PHPT patients over the past 12 years (Table 3). A total of 177 and 508 patients were diagnosed with PHPT during these two time periods, respectively. There were no significant differences in gender, age, disease duration, or BMI between the two groups.

**Table 3:** A comparison of clinical features between group A (2013–2018) and group B (2019–2024).

	Group A (2013–2018)	Group B (2019–2024)	*p*
Number (%)	177 (25.8)	508 (74.2)	
Gender (male/female) (*n*/%)	42 (23.7)/135 (76.3)	124 (24.4)/384 (75.6)	0.856
Age (years)	60.0 (52.0, 67.0)	59.0 (50.0, 66.0)	0.206
Age < 50 years (*n*/%)	33 (18.6)	125 (24.6)	0.105
Disease duration (months)	12.0 (1.0, 60.0)	6.0 (1.0, 24.0)	0.081
BMI (kg/m^2^)	23.39 (21.06, 26.73)	24.03 (21.92, 26.04)	0.508
PTH (1.1–7.3 pmol/L)	31.80 (19.45, 73.35)	19.80 (12.73, 34.33)	**< 0.001**
Serum calcium (2.15–2.55 mmol/L)	2.75 (2.61, 2.90)	2.75 (2.63, 2.98)	0.113
Serum phosphate (0.80–1.45 mmol/L)	0.80 (0.65, 0.95)	0.82 (0.70, 0.93)	0.526
Urine calcium (2.5–7.5 mmol/24 h)	8.75 (5.71, 10.62)	7.88 (5.66, 10.40)	0.153
Urine phosphate (23–48 mmol/24 h)	18.60 (13.20, 22.84)	19.71 (15.10, 25.73)	0.008
eGFR (ml/min ∗ 1.73 m^2^)	117.96 (86.85, 154.36)	120.21 (96.14, 146.64)	0.903
Urine pH (5.5–8.0)	6.0 (6.0, 6.5)	6.0 (5.5, 7.0)	0.422
25‐OHD (15.5–133.0 nmol/L)	29.14 (24.23, 39.73)	28.06 (20.04, 38.60)	0.044
ALKP (40–150 U/L)	91.0 (70.0, 133.0)	107.0 (77.0, 153.0)	0.036
OC (11.0–48.0 ng/mL)	43.09 (27.45, 84.88)	41.09 (27.59, 68.08)	0.186
CTX (0.30–1.01 ng/mL)	1.08 (0.56, 1.74)	1.04 (0.62, 1.55)	0.634
PINP (19–84 ng/mL)	70.59 (43.80, 127.64)	80.95 (52.42, 128.05)	0.332
BMD_L1–L4_ (g/cm^2^)	0.908 (0.798, 1.035)	0.942 (0.783, 1.066)	0.269
BMD_femoral neck_ (g/cm^2^)	0.712 (0.625, 0.825)	0.725 (0.629, 0.818)	0.780
BMD_total hip_ (g/cm^2^)	0.757 (0.651, 0.869)	0.772 (0.651, 0.873)	0.759
Conscious symptoms	138 (78.0)	326 (64.2)	**0.001**
First symptoms			
Bone	72 (40.7)	159 (31.3)	**0.023**
Muscle	58 (32.8)	124 (24.4)	**0.030**
Digestive tract	68 (38.4)	105 (20.7)	**< 0.001**
Urinary system	70 (39.5)	134 (26.4)	**0.001**
Neuropsychiatric system	32 (18.1)	33 (6.5)	**< 0.001**
Surgical treatment	75 (42.4)	342 (67.3)	**< 0.001**
Bone complications			
None	82 (46.3)	235 (46.3)	**< 0.001**
Osteopenia	13 (7.3)	105 (20.7)^∗^	
Osteoporosis	82 (46.3)	168 (33.1)^∗^	
Nephrolithiasis	68 (38.4)	200 (39.4)	0.823

*Note:* The intergroup comparisons were conducted using the Mann–Whitney *U* test. Since 18 continuous indicators were compared simultaneously, the Bonferroni method was used for correction. After correction, the significance level *α* ^′^ = 0.003. The *p* values in the table are all the original values. However, the differences are considered statistically significant only when the original *p* value of the continuous indicators is less than 0.003 (i.e., for PTH, *p* < 0.001). The significance of categorical variables is still based on the criterion of *p* < 0.05. Bold *p* values indicate statistical significance.

Abbreviations: 25‐OHD = 25 hydroxyvitamin D, ALKP = alkaline phosphatase, BMD = bone mineral density, BMI = body mass index, CTX = Type I collagen carboxy‐terminal peptide, eGFR = estimated glomerular filtration rate, OC = osteocalcin, PINP = Type I procollagen amino‐terminal prepeptide, PTH = parathyroid hormone.

^∗^After pairwise comparisons in the Chi‐square test, the difference was statistically significant when compared with Group A, adjusted *p* < 0.05.

As shown in Table 3, after performing the Mann–Whitney *U* test and applying Bonferroni correction (*α* ^′^ = 0.003), it was found that the level of PTH in group B was significantly lower than that in group A (*p* < 0.001), while there was no statistically significant difference in the level of urine phosphate between the two groups (*p* = 0.008 > 0.003).

Compared with group A, a lower percentage of patients in group B had conscious symptoms (78.0 vs. 64.2%; *p* < 0.01), which meant the proportion of asymptomatic patients increased from 22.0% in group A to 35.8% in group B. Moreover, 72 patients in group A complained of bone symptoms, a higher percentage than in group B (40.7 vs. 31.3%; *p* < 0.05), and muscle fatigue or pain, digestive tract, urinary system, and neuropsychiatric system symptoms were much more common in group A than those in group B (32.8 vs. 24.4%; 38.4 vs. 20.7%; 39.5 vs. 26.4%; 18.1 vs. 6.5%; *p*_*a* *l* *l*_ < 0.05). The proportion of surgical treatment was higher in group B (42.4 vs. 67.3%; *p* < 0.01). The younger age profile in asymptomatic patients of group B, with 28.0% of patients under 50 years old, was higher than that in group A (20.5%, data not shown in Table 1). Although no significant differences were found between two groups in statistics in BMD, the percentage of osteoporosis in group B was lower than that in group A (46.3 vs. 33.1%; *p* < 0.05).

### 3.3. Comparison of Asymptomatic and Symptomatic Patients

We compared asymptomatic patients (221 cases, 32.3%) and symptomatic patients (464 cases, 67.7%). No significant differences in gender and BMI were found between the two groups. Symptomatic patients had longer disease duration than asymptomatic patients. When it comes to laboratory tests, symptomatic patients had significantly higher serum PTH, calcium, OC, and urine pH levels than those in asymptomatic patients while with lower serum phosphate than that in asymptomatic patients (Table 4). Symptomatic patients had worse bone condition than asymptomatic patients, with lower BMD in L1–L4 (*p* < 0.003). After adjustment for age, gender, and BMI, significant positive associations were observed between the symptomatic PHPT and disease duration, PTH, serum calcium, and OC levels. In contrast, inverse associations were found with serum phosphate and BMD in L1–L4. The adjusted ORs are presented in Supporting Table 1.

**Table 4:** A comparison of clinical features between asymptomatic and symptomatic PHPT.

	Asymptomatic group	Symptomatic group	*p*
Number (%)	221 (32.3)	464 (67.7)	
Gender (male/female) (*n*/%)	54 (24.4)/167 (75.6)	112 (24.1)/352 (75.9)	0.933
Age (years)	57.0 (49.0, 65.0)	60.0 (51.0, 67.0)	0.018
Age < 50 years (*n*/%)	59 (26.7)	99 (21.3)	0.122
Disease duration (months)	2.0 (0.5, 12.0)	12.0 (2.0, 57.0)	**< 0.001**
BMI (kg/m^2^)	23.92 (21.64, 26.03)	24.02 (21.90, 26.04)	0.819
PTH (1.1–7.3 pmol/L)	18.00 (11.60, 26.58)	26.45 (16.10, 59.48)	**< 0.001**
Serum calcium (2.15–2.55 mmol/L)	2.69 (2.59, 2.84)	2.79 (2.65, 3.00)	**< 0.001**
Serum phosphate (0.80–1.45 mmol/L)	0.85 (0.75, 0.95)	0.79 (0.65, 0.92)	**< 0.001**
Urine calcium (2.5–7.5 mmol/24 h)	8.02 (5.05, 10.25)	8.19 (5.76, 10.56)	0.284
Urine phosphate (23–48 mmol/24 h)	20.43 (16.13, 26.02)	18.96 (13.81, 24.65)	0.009
eGFR (ml/min ∗ 1.73 m^2^)	122.78 (99.23, 148.26)	117.96 (90.48, 149.70)	0.163
Urine pH (5.5–8.0)	6.0 (5.5, 6.5)	6.5 (6.0, 7.0)	**0.001**
25‐OHD (15.5–133.0 nmol/L)	28.53 (20.61, 41.34)	28.34 (21.19, 37.56)	0.379
ALKP (40–150 U/L)	94.0 (74.0, 132.0)	107.0 (76.0, 157.3)	0.022
OC (11.0–48.0 ng/mL)	36.54 (26.41, 57.09)	46.71 (28.35, 82.74)	**< 0.001**
CTX (0.30–1.01 ng/mL)	0.98 (0.61, 1.38)	1.07 (0.62, 1.76)	0.076
PINP (19–84 ng/mL)	78.11 (51.36, 118.35)	81.28 (47.12, 134.09)	0.358
BMD_L1–L4_ (g/cm^2^)	0.962 (0.865, 1.124)	0.909 (0.763, 1.041)	**0.001**
BMD_femoral neck_ (g/cm^2^)	0.750 (0.659, 0.846)	0.703 (0.612, 0.811)	0.005
BMD_total hip_ (g/cm^2^)	0.790 (0.700, 0.890)	0.754 (0.612, 0.859)	0.003
Surgical treatment	127 (57.5)	290 (62.5)	0.207
Bone complications			
None	126 (57.0)	191 (41.2)^∗^	**< 0.001**
Osteopenia	46 (20.8)	72 (15.5)	
Osteoporosis	49 (22.2)	201 (43.3)^∗^	
Nephrolithiasis	50 (22.6)	218 (47.0)	**< 0.001**

*Note:* The intergroup comparisons were conducted using the Mann–Whitney *U* test. Since 18 continuous indicators were compared simultaneously, the Bonferroni method was used for correction. After correction, the significance level *α* ^′^ = 0.003. The *p* values in the table are all the original values. However, the differences are considered statistically significant only when the original *p* value of the continuous indicators is less than 0.003 (i.e., for PTH, *p* < 0.001). The significance of categorical variables is still based on the criterion of *p* < 0.05. Bold *p* values indicate statistical significance.

Abbreviations: 25‐OHD = 25 hydroxyvitamin D, ALKP = alkaline phosphatase, BMD = bone mineral density, BMI = body mass index, CTX = type I collagen carboxy‐terminal peptide, eGFR = estimated glomerular filtration rate, OC = osteocalcin, PINP = type I procollagen amino‐terminal prepeptide, PTH = parathyroid hormone.

^∗^After pairwise comparisons in the Chi‐square test, the difference was statistically significant when compared with the asymptomatic group, adjusted *p* < 0.05.

Compared with asymptomatic patients, the percentage of osteoporosis was higher (22.2 vs. 43.3%; *p* < 0.05) and there were more patients suffering from nephrolithiasis in symptomatic patients (22.6 vs. 47.0%; *p* < 0.01).

### 3.4. Comparison of Patients With Benign, Atypical, and Malignant Parathyroid Tumors

The group of benign parathyroid tumors includes both multiglandular parathyroid diseases and parathyroid adenomas. Compared with benign cases, atypical cases and malignant cases presented higher serum PTH and calcium levels and lower serum phosphate level (adjusted *p* < 0.05). Moreover, serum ALKP and urine calcium levels also had higher tendency in atypical cases than those in benign cases (adjusted *p* < 0.05) (Table 5). After adjustment for age, gender, and BMI, significant positive associations were observed between the nonbenign pathological type of PHPT and PTH and serum calcium levels. In contrast, inverse associations were found with serum phosphate. The adjusted ORs are presented in Supporting Table 2.

**Table 5:** Clinical and biochemical characteristics of benign, atypical, malignant parathyroid tumor cases.

	Benign	Atypical	Malignant	*p*
Number (%)	373 (89.4)	34 (8.2)	10 (2.4)	
Gender (male/female) (*n*/%)	82 (22.0)/291 (78.0)	10 (29.4)/24 (70.6)	5 (50.0)/5 (50.0)	0.111
Age (years)	58.0 (49.5, 65.0)	55.0 (43.8, 62.0)	55.5 (41.0, 69.0)	0.219
Age < 50 years (*n*/%)	93 (24.9)	12 (35.3)	4 (40.0)	0.276
Disease duration (months)	6.0 (1.0, 36.0)	12.0 (2.0, 60.0)	12.0 (2.0, 54.0)	0.414
BMI (kg/m^2^)	23.80 (21.70, 25.94)	24.62 (21.17, 26.28)	23.42 (22.51, 25.27)	0.965
PTH (1.1–7.3 pmol/L)	22.70 (15.40, 40.90)	54.42 (20.05, 147.75)^∗^	70.41 (36.78, 120.0)^∗^	< 0.001
Serum calcium (2.15–2.55 mmol/L)	2.77 (2.64, 2.98)	2.96 (2.73, 3.31)^∗^	3.05 (2.96, 3.23)^∗^	< 0.001
Serum phosphate (0.80–1.45 mmol/L)	0.81 (0.68, 0.92)	0.74 (0.62, 0.80)^∗^	0.57 (0.49, 0.75)^∗^	0.001
Urine calcium (2.5–7.5 mmol/24 h)	8.06 (5.73, 10.34)	10.18 (7.03, 13.12)^∗^	9.15 (6.65, 11.82)	0.028
Urine phosphate (23–48 mmol/24 h)	19.47 (14.88, 25.00)	19.00 (12.12, 26.36)	23.07 (10.28, 33.99)	0.672
eGFR (ml/min ∗ 1.73 m^2^)	124.17 (98.93, 151.37)	121.71 (81.17, 150.16)	102.54 (56.90, 137.36)	0.203
Urine pH (5.5–8.0)	6.0 (5.5, 6.5)	6.5 (5.9, 7.0)	6.3 (5.5, 7.0)	0.659
25‐OHD (15.5–133.0 nmol/L)	27.03 (20.82, 35.31)	24.16 (19.34, 37.29)	28.86 (17.04, 32.36)	0.759
ALKP (40–150 U/L)	109.5 (78.0, 156.0)	145.0 (97.0, 225.5)^∗^	141.0 (110.0, 234.5)	0.010
OC (11.0–48.0 ng/mL)	46.27 (30.45, 76.47)	59.70 (35.25, 190.95)	107.90 (57.69, 137.95)	0.020
CTX (0.30–1.01 ng/mL)	1.13 (0.66, 1.69)	1.36 (0.94, 2.54)	1.35 (1.04, 2.33)	0.103
PINP (19–84 ng/mL)	94.77 (53.35, 139.05)	105.50 (68.55, 230.15)	95.06 (70.34, 181.50)	0.171
Conscious symptoms	260 (69.7)	24 (70.6)	6 (60.0)	0.806
First symptoms				
Bone	129 (34.6)	13 (38.2)	2 (20.0)	0.542
Muscle	97 (26.0)	7 (20.6)	3 (30.0)	0.741
Digestive tract	95 (25.5)	11 (32.4)	2 (20.0)	0.628
Urinary system	122 (32.7)	13 (38.2)	2 (20.0)	0.534
Neuropsychiatric system	34 (9.1)	1 (2.9)	1 (10.0)	0.374
Bone complications				
None	176 (47.2)	13 (38.2)	7 (70.0)	0.391
Osteopenia	64 (17.2)	9 (26.5)	1 (10.0)	
Osteoporosis	133 (35.7)	12 (35.3)	2 (20.0)	
Nephrolithiasis	160 (42.9)	19 (55.9)	3 (30.0)	0.232

*Note:* The intergroup comparisons were conducted using the Kruskal–Wallis *H* test. Since the overall test was significant, pairwise comparisons were further carried out, and the *p* values were corrected using the Holm‐Bonferroni method.

Abbreviations: 25‐OHD = 25 hydroxyvitamin D, ALKP = alkaline phosphatase, BMD = bone mineral density, BMI = body mass index, CTX = type I collagen carboxy‐terminal peptide, eGFR = estimated glomerular filtration rate, OC = osteocalcin, PINP = type I procollagen amino‐terminal prepeptide, PTH = parathyroid hormone.

^∗^The difference was statistically significant when compared with benign tumor, adjusted *p* < 0.05.

However, no differences were observed among patients with benign and atypical tumors and carcinoma in terms of gender, age, disease duration, BMI, urine phosphate, eGFR, urine pH, serum 25‐OHD or conscious symptoms, first symptoms, bone complications, and nephrolithiasis.

## 4. Discussion

In this study, we analyzed 685 PHPT patients, elucidating the evolving clinical and biochemical characteristics over a 12 year period (2013–2024), particularly the rising incidence of both symptomatic and asymptomatic cases. Notably, the number of PHPT cases in 2024 was nearly six times higher than that in 2013, and patients diagnosed between 2021 and 2024 accounted for over 60% of all cases during the study period. This trend may reflect increased awareness of osteoporosis assessment, leading to more frequent serum PTH and calcium testing.

Comparing two time periods, patients in group B (2019–2024) presented with significantly lower serum PTH levels and milder symptoms than those in group A (2013–2018). Earlier diagnosis and timely treatment in group B likely contributed to a higher surgical rate and lower prevalence of osteoporosis. The younger age distribution in group B may have further facilitated surgical intervention, despite a higher prevalence of asymptomatic cases. Moreover, the number of asymptomatic patients in 2024 increased more than tenfold compared with 2013. Unlike Western populations, where asymptomatic PHPT accounts for 80%–90% of cases [11, 12], our study observed a proportion of 32.3%, with a steadily rising trend over the years, consistent with other reports [13, 14].

The most common systemic symptoms in our cohort involved the bones, urinary system, and muscles, consistent with previous studies [4, 5, 13–15]. Nearly half (46.8%) of symptomatic patients sought medical attention due to bone pain, 37.3% were diagnosed with nephrolithiasis, and 36.2% experienced fatigue. Interestingly, gastrointestinal symptoms, particularly nausea and vomiting, were reported in 35.1% of patients, higher than in other studies. This may reflect a deeper clinical awareness of subtle PHPT‐related manifestations, leading clinicians to investigate previously overlooked symptoms.

Most asymptomatic cases in our study were identified through routine serum calcium testing, highlighting the increasing use of multichannel biochemical analysis in daily clinical practice over the past decade [13]. Additionally, 46.9% of asymptomatic patients presented due to parathyroid nodules or adenomas incidentally detected by neck ultrasonography. Given the high prevalence of thyroid nodules [16], routine neck/thyroid ultrasonography has contributed to the increasing incidental detection of PHPT.

Comparing symptomatic and asymptomatic patients, we observed that asymptomatic individuals generally had lower PTH levels, milder disease manifestations, and preserved bone density, in line with previous research [13]. Skeletal involvement remains a hallmark of PHPT, as excess PTH increases bone resorption and remodeling, reflected by elevated bone turnover markers (BTMs) [17, 18]. Our results indeed demonstrated a significant decrease in BMD at all measured sites in symptomatic patients. A closer comparison reveals that the bone loss was more pronounced at the lumbar spine compared to the hip regions. This apparent discrepancy—where the predominantly trabecular lumbar spine shows greater BMD loss—seems to contradict the traditional view that PHPT primarily affects cortical bone [19, 20]. However, this very observation aligns with the evolving understanding of PHPT’s impact on bone microstructure. As noted in the literature [21], while PTH‐mediated remodeling thins cortical bone, it also profoundly affects trabecular architecture by thinning trabeculae and increasing porosity. The notion of “trabecular preservation” in PHPT has been challenged by advanced assessment tools like the trabecular bone score (TBS), which reveal significant degradation of trabecular microarchitecture despite potentially preserved areal BMD [21].

Symptomatic patients with higher urine pH were more likely to develop nephrolithiasis, likely due to increased formation of calcium phosphate stones at urine pH > 6.5 [22–24]. Nevertheless, not all asymptomatic patients were free of target organ damages; some demonstrated low BMD on DXA or nephrolithiasis on ultrasonography [20], highlighting that these individuals may still meet indications for PTX [6, 20]. Surgical treatment has been shown to achieve biochemical cure and improve BMD in both symptomatic and selected asymptomatic patients [25–27]. For asymptomatic patients who do not meet surgical criteria, predictive models have been proposed to guide optimal timing for intervention [6].

Preoperative diagnosis of parathyroid carcinoma remains challenging due to overlapping clinical features with benign PHPT. Typically, malignant cases present with serum PTH levels 5–10 times above the upper limit of normal, whereas benign PHPT usually shows PTH levels up to twice the upper limit [28, 29]. In our study, carcinoma cases exhibited PTH values approximately tenfold above the normal range and were associated with more severe biochemical abnormalities, including higher serum calcium and lower phosphate levels.

This study has several limitations. Its retrospective design introduces potential selection bias, which may affect the generalizability of findings. Limited longitudinal follow‐up may obscure the progression of clinical and biochemical changes over time. Furthermore, exclusion of outpatient PHPT cases may have led to underestimation of asymptomatic patient prevalence. Future studies incorporating outpatient data and prospective follow‐up are warranted to capture the full spectrum of PHPT and validate these findings.

In conclusion, our research delineates the clinical and biochemical features of PHPT, highlighting a rising prevalence of asymptomatic cases, while symptomatic presentations remain common. Parathyroid carcinoma exhibits more severe clinical and biochemical characteristics than benign PHPT. These findings underscore the need for heightened awareness of asymptomatic PHPT and a more proactive approach to diagnosis and management, emphasizing the importance of early detection and appropriate intervention.

## Disclosure

All authors reviewed and approved the final manuscript.

## Conflicts of Interest

The authors declare no conflicts of interest.

## Author Contributions

Ming Liu and Yuansi Chen contributed to the study conception and design. Material preparation, data collection, and analysis were performed by Yuansi Chen, Junhe Wang, and Shuo Li. Qinying Zhao, Kunling Wang, and Ming Liu discussed and edited the manuscript. Yuansi Chen, Junhe Wang, and Shuo Li have contributed equally to this work and share first authorship.

## Funding

This work was funded by Tianjin Key Medical Discipline (Specialty) Construction Project (TJYXZDXK‐030A) and Tianjin Medical University Clinical Special Disease Research Center‐Neuroendocrine Tumor Clinical Special Disease Research Center.

## Supporting Information

Supporting Table 1 shows the adjusted ORs in multivariate regression analysis of factors influencing the presence of symptoms in patients with PHPT. Supporting Table 2 shows the adjusted ORs in multivariate regression analysis of factors influencing the pathological type in patients with PHPT.

## Supporting information


**Supporting Information** Additional supporting information can be found online in the Supporting Information section.

## Data Availability

Some or all datasets generated during and/or analyzed during the current study are not publicly available but are available from the corresponding authors on reasonable request.
