# Supplementary material for: Trends in Clinical Features of Primary Hyperparathyroidism During 2013–2024
Source: Int J Endocrinol. 2026 Jan 7;2026:9936658. doi: 10.1155/ije/9936658 (PMC12776255; doi:10.1155/ije/9936658)
Supplement: Supplementary file 1 — Supporting Information Additional supporting information can be found online in the Supporting Information section. [file IJE-2026-9936658-s001.docx]

**Supplementary Table 1** Multivariate regression analysis of factors influencing the presence of symptoms in patients with PHPT

| **Variables** | ***β*** | **S.E.** | **Odds ratio(95%CI)** | ***P*** |
| --- | --- | --- | --- | --- |
| Disease duration | 0.015 | 0.004 | 1.015(1.008,1.022) | **<0.001** |
| PTH | 0.016 | 0.004 | 1.016(1.008,1.024) | **<0.001** |
| Serum calcium | 1.919 | 0.448 | 6.816(2.833,16.395) | **<0.001** |
| Serum phosphate | -2.685 | 0.623 | 0.068(0.020,0.231) | **<0.001** |
| Urine pH | 0.232 | 0.145 | 1.261(0.950,1.674) | 0.109 |
| OC | 0.008 | 0.003 | 1.008(1.003,1.014) | **0.001** |
| BMD _L1-L4_ | -2.067 | 0.878 | 0.127(0.023,0.708) | **0.019** |

Note: Abbreviations are the same as which in Table 3. *β*: regression coefficient. The presence of symptoms in patients with PHPT was the dependent variable (y=0 meant asymptomatic, y=1 meant symptomatic). The analysis of each independent variable adjusted age, gender, and BMI. Bolded P-values indicate statistical significance.

**Supplementary Table 2** Multivariate regression analysis of factors influencing the pathological type in patients with PHPT

| **Variables** | ***β*** | **S.E.** | **Odds ratio(95%CI)** | ***P*** |
| --- | --- | --- | --- | --- |
| PTH | 0.008 | 0.003 | 1.008(1.002,1.013) | **0.007** |
| Serum calcium | 2.080 | 0.602 | 8.001(2.460,26.026) | **0.001** |
| Serum phosphate | -3.524 | 1.182 | 0.029(0.003,0.299) | **0.003** |
| Urine calcium | 0.035 | 0.045 | 1.036(0.948,1.132) | 0.434 |
| ALKP | 0.001 | 0.000 | 1.001(1.000,1.002) | 0.257 |

Note: Abbreviations are the same as which in Table 3. *β*: regression coefficient. The pathological type in patients with PHPT was the dependent variable (y=0 meant benign, y=1 meant non-benign). The analysis of each independent variable adjusted age, gender, and BMI. Bolded P-values indicate statistical significance.
